# A triaged real-time alert intervention to improve antiretroviral therapy adherence among young African American men who have sex with men living with HIV: focus group findings

**DOI:** 10.1186/s12889-019-6689-1

**Published:** 2019-04-11

**Authors:** Mark S. Dworkin, Palak Panchal, Wayne Wiebel, Robert Garofalo, Jessica E. Haberer, Antonio Jimenez

**Affiliations:** 1 Division of Epidemiology and Biostatistics, University of Illinois at Chicago School of Public Health, 1603 W. Taylor Street, MC 923, Chicago, IL 60612 USA; 2 Department of Pediatrics, Northwestern University/Ann & Robert H. Lurie Children’s Hospital of Chicago, Chicago, IL USA; 3 Massachusetts General Hospital Center for Global Health, Boston, MA 02114 USA; 4 University of Illinois at Chicago School of Public Health, Community Outreach Intervention Projects, 1603 W. Taylor Street, Chicago, IL 60612 USA

**Keywords:** Real-time, Adherence, HIV, Antiretroviral, Men who have sex with men, Wisepill, Monitoring, Text message

## Abstract

**Background:**

Among persons living with HIV, poorer antiretroviral therapy adherence has been reported in African Americans and disproportionate mortality reported in young African American men who have sex with men (AAMSM) compared to whites. We report the results of focus groups with young AAMSM living with HIV that explore their opinions about the acceptability and feasibility of a triaged real-time missed dose alert intervention to improve treatment adherence.

The purpose of this study is to develop a theory-driven triaged real-time adherence monitoring intervention to promote HIV medication adherence in young AAMSM.

**Methods:**

We performed five focus groups and two individual interviews among young HIV-positive AAMSM (*n* = 25) in Chicago guided by the Technology Acceptance Model and explored perceptions regarding the monitoring concept including device issues and concerns about inclusion of support persons whose involvement is triggered by sustained missed doses. The purpose was to inform the development of this intervention in this population.

**Results:**

Generally, the participants found the proposed intervention acceptable and useful. Privacy was a major concern for participants especially with attention to possible disclosure of their HIV status by receiving a medication-related text that someone else might view and could lead to unwanted attention. There was concern that the device could be confused with a taser. Approximately half of the men already had a close personal contact that helped them with medication taking. Some participants acknowledged that the notification might lead to friction.

**Conclusions:**

A triaged real-time alert intervention to improve treatment adherence is acceptable and feasible among young AAMSM living with HIV.

## Introduction

Young African American men who have sex with men (AAMSM) are disproportionately impacted by new HIV infections and HIV-related mortality [1–5]. MSM account for more than three-fourths of all new HIV infections among men in the U.S. and nearly half of these are AAMSM [1]. Maintaining optimal antiretroviral adherence reduces morbidity, mortality, emergence of resistant virus, and risk for HIV transmission to others [4]. Yet, a large number of persons living with HIV do not achieve or maintain adherence levels necessary for viral suppression. Although better adherence has been observed with newer regimens of antiretroviral therapy (ART) compared to the first decade of highly active ART [6–13], racial disparities exist with several studies demonstrating poorer adherence among African Americans compared to whites [14–20]. A report of pooled data from 13 studies revealed the odds of 100% adherence were 40% lower in African Americans than whites. In a study of HIV-positive patients on ART in Chicago, African American patients were twice more likely than whites to be virally detectable [21].

There are many barriers and facilitators to ART adherence and ART adherence interventions [22, 23]. One of the more common barriers is forgetting. Some studies have demonstrated that interventions that include text messaging or reminder alerts may be effective at overcoming this problem [24–26]. This approach may be especially helpful for persons who lack a close trusted friend, lover, or family member since an important facilitator of adherence is social support. Studies have demonstrated that social support among people living with HIV infection is associated with better health outcomes [27–31] and adherence to treatment [30, 32–39]. Social support may be received in several different forms including providing empathy (emotional support), information (informational support), or tangible services (instrumental support) [40]. These forms of support have been described by MSM living with HIV across all stages of the HIV continuum of care and may facilitate adherence by improving mental health, increased HIV knowledge, and accountability to a trusted close contact [41].

Recently, real-time adherence detection has been employed as an adherence intervention. Haberer and colleagues performed a pilot randomized controlled trial in Uganda with 62 HIV-positive participants recruited from a hospital in a largely rural area. Thirty-seven percent of these study participants reported severe food sinsecurity [42]. Adherence was 11.1% higher for the study arm that employed a combination of initially scheduled text message reminders followed by reminders that were triggered by a late or missed dose and text message notification to social supporters for adherence lapses > 48 h compared to the control group. However adherence was generally high for both this intervention arm and the control group (median adherence 92% and 90%, respectively). Sabin and colleagues performed a randomized clinical trial with 120 HIV patients in China who were provided personalized triggered real-time reminders when missed doses were detected, coupled with monthly adherence counseling when suboptimal adherence was identified [43]. They found that the intervention group was 2.4 times more likely than controls to achieve optimal (> 95%) adherence. Orrell and colleagues performed a randomized controlled trial with 230 participants in South Africa [44]. Compared to standard of care with three pretreatment education sessions, an intervention that included standard of care and automated text reminders in response to late doses (> 30 min) detected in real-time had a median adherence of 82.1% compared to 80.4% and reduced treatment interruptions of > 72 h. Two studies in the US have explored real-time adherence detection as part of an intervention to improve adherence. Pellowski and colleagues studied feasibility and acceptability of an intervention that included real-time counseling and motivation by phone in 21 men and women living with HIV in Atlanta within a 41 participant study [45] The employment of the device and counseling was considered acceptable. However, nearly half of the participants were uncomfortable about being monitored. Stringer and colleagues studied feasibility and acceptability of real-time adherence monitoring among 25 depressed African American women living with HIV in four rural communities in Alabama, many of whom were living in poverty [46]. Generally, real-time adherence monitoring was considered acceptable and feasible. Participants reported that they felt a sense of accountability, which may have motivated them towards better adherence. Although they experienced some technical failures of the device that delayed transmission of adherence, only 5.7% of expected events were lost. We are unaware of any studies of real-time adherence as an intervention in the US or globally that focused on an MSM population.

Here we report the results of focus groups with young AAMSM living with HIV in Chicago that explore their opinions about the acceptability and feasibility of such a triaged real-time missed dose alert intervention to improve treatment adherence. To our knowledge, there are no studies looking at feasibility, acceptability, or effectiveness of real-time adherence detection strategies among AAMSM. This is the first paper to report opinions about using real-time monitoring as an adherence intervention in young AAMSM.

## Methods

### Intervention

**Fig. 1:**
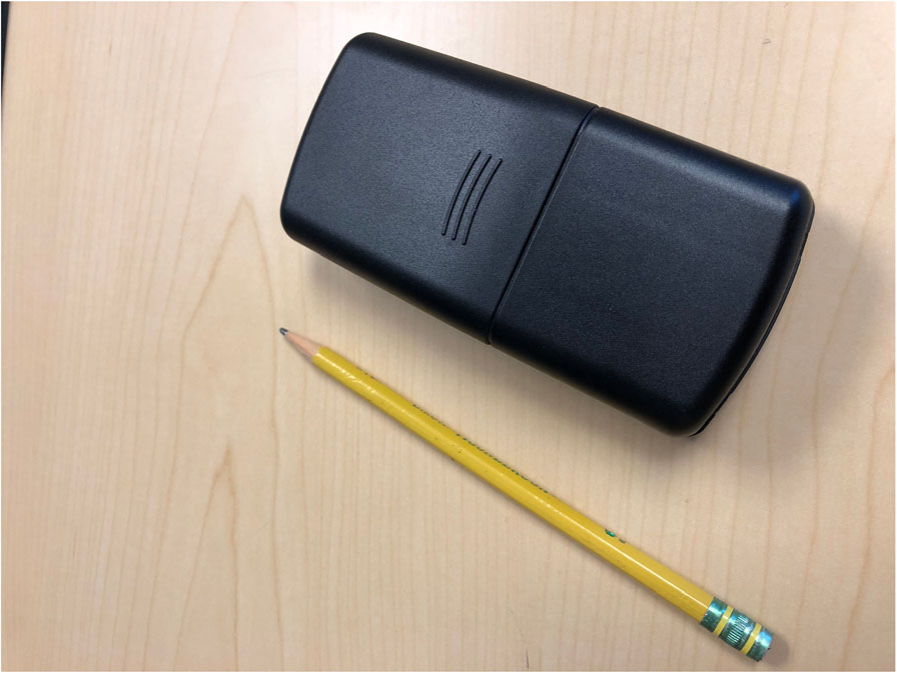
Wisepill device used for electronic adherence monitoring

**Fig. 2:**
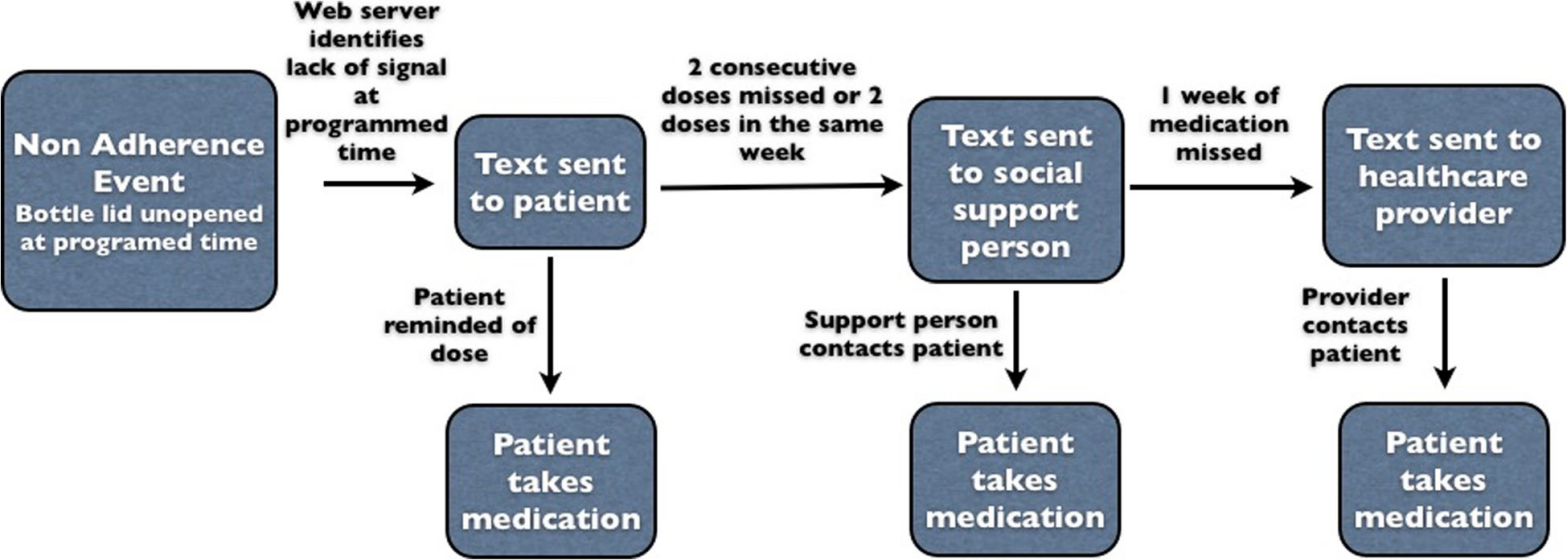
Proposed intervention scheme where a text message alert of a missed dose can be sent to either a patient, their social support person, or a healthcare provider or case manager depending on the duration of number of days a dose has been missed

We propose an intervention to improve ART adherence for young AAMSM living with HIV that employs a triaged approach, utilizes real-time adherence monitoring, and leverages existing social support. By triaged approach, we mean that this intervention will provide different types of back-up support depending on the duration of real-time recognized missed doses. This intervention is grounded in the information, motivation, behavioral skills (IMB) model of antiretroviral therapy adherence that focuses on feedback between the information and motivation that affect one’s behavioral skills, behaviors, and desired health outcomes [47]. In our proposed intervention, information about and motivation for treatment adherence may be received or reinforced from text messages, a social support person, and a case manager, all of which are expected to improve adherence and viral load suppression. We hypothesize that this approach, which attempts to intervene with triaged support, will lead to improved adherence in patients with suboptimal adherence. Electronic real-time nonadherence monitoring can be performed using a device such as Wisepill (Fig. 1), a pill container monitoring device that uses an embedded global mobile communications chip to capture device openings, as a proxy for adherence, in real-time by sending a signal to a central server at each opening (Wisepill, Capetown, South Africa) [48]. The server is provided with cell phone contact information in order that a responsive text message can then be sent automatically when doses are missed, informing the user so that an overdue dose may be taken later the same day (thus, ideally eliminating the miss). If two consecutive doses are missed, a social support person designated by the user will be alerted, which may lead to a text, phone call, or in-person reminder intended to motivate the user’s adherence. Finally, if seven consecutive days are missed, a previously designated case manager or other healthcare provider will receive the alert and contact the user to investigate the situation. Based upon what is found, they may strategize to resolve adherence issues, such as insurance lapse or need for referral to substance use or mental health counseling (Fig. 2).

Study procedures and participants.

In order to inform intervention development, five focus groups with 3 to 7 participants in each group (*N* = 25) and two individual discussions were conducted in Chicago during December 2016 through January 2018.

Participants for the focus groups were recruited from four University of Illinois at Chicago Community Outreach Intervention Project (COIP) sites located in high HIV incidence areas of the city and the University of Illinois at Chicago HIV clinic using fliers and word of mouth. Inclusion criteria for the study included being age 18–34 years, African American, MSM, living with HIV, on ART for at least 3 months by self-report, and having a detectable viral load in the past 12 months. Three out of 25 participants provided the viral load reports to us and these were detectable.

All procedures were approved by the University of Illinois at Chicago School of Public Health Institutional Review Board. Before each focus group began, informed consent was obtained. Focus groups were performed in a confidential setting either at the UIC School of Public Health or a COIP site and were led by an experienced focus group moderator. Participants were encouraged not to share what was discussed within the room or disclose anyone else’s participation (full names were not used). Discussion guidelines sought to explore participant’s perceptions of the usefulness, convenience, concerns about, and willingness to participate in a triaged real-time text alert nonadherence intervention. One-on-one interviews followed the same protocol as the focus groups and were performed in the two instances that only one participant showed up for a focus group. Focus groups lasted up to 2 h and were audio-recorded and later transcribed for data analysis. The data were sent out for professional transcription service, babble type. Subjects were paid $50 compensation for expenses and participation.

#### Interview structure

Before the start of focus groups, participants were provided a short questionnaire to determine their demographic characteristics, health information, ability to read, if they currently had or could identify a person who supports or could support their ART adherence, lifetime and past 6-month history of illicit substances, alcohol consumption, and HIV treatment history including duration of ART and self-reported 4-day adherence. Participants were also asked about three adherence facilitators using a Likert scale including communication with their healthcare provider, how comfortable they are with their knowledge about ART, and how comfortable they are with their knowledge of possible ART side effects.

An interview guide was developed and utilized to organize focus groups discussions. The guide followed recommendations of the Technology Acceptance Model [49, 50]. In this model, the perceived usefulness and perceived ease of use of technology affects one’s attitude and that affects intent and actual use of the technology. Therefore, we included questions about whether the participants perceived the proposed intervention as useful and if they perceived the Wisepill device as easy to use. At the beginning of the focus group, the proposed intervention was described and the Wisepill device was passed around the room. Commentary included participant perceptions regarding the monitoring concept (i.e., its usefulness and concern for being “tracked”), convenience/ease of use, practicality vis-a-vis size, storage, handling, related cell phone practices, and intention to use. They were asked about how they avoid missing medication when they are away from home because although the Wisepill device is small and transportable, a few pills can be removed and taken along (as when planning a sleepover or travel) just like with an ordinary pill bottle. They were also asked their viewpoints regarding contact identification (whether a doctor or case manager should be notified of a 7-consecutive day miss), perceptions about messaging procedures, and feasibility in varied settings/situations. Because the proposed intervention includes a triaged approach with designation of a social support person (if they have one) to receive a text message when nonadherence duration reaches a certain threshold, they were asked about perceptions on the role of the patient-chosen support person and characteristics influencing their suitability (e.g., to what extent they are a “support” and if they are concerned how involvement of the contact might affect their relationship). In addition, participants were asked about preferences for the nonadherence responsive messaging (specifically, what do they want it to say ranging from a symbol to an educational and self-efficacy promoting statement). Finally, they were asked about device design features including possible concerns about privacy or stigma if someone else saw or found the device.

#### Data analysis

Data from the demographic/health background questionnaires were analyzed descriptively. For the five focus groups and two one-on-one interviews, team members reviewed transcripts of participant discussions and narrative passages were coded based on topical content. Analysis involved open coding, axial coding, and selective coding [51–53]. Themes were determined that might yield important and interesting information to inform the proposed intervention. We used QDA Miner Lite software to systematically code connecting blocks of text. The software analysis was performed by one member of the research team and all coded output were reviewed by the other three members.

## Results

### Participant characteristics

Demographic characteristics and health information from the 25 participants are summarized in Table 1. The median age was 29 years. The median number of doses missed in the past 4 days was 1 (range 0–6). Among the 12 men who did not have a close personal contact who currently helped them remember to take their medication (such as a family member or partner), 4 (33%) did have someone they could ask to help in this way if they needed help. For the 13 men who had at least one close personal contact currently helping them, these support persons were a mother (23%), father (8%), brother (15%), sister (8%), partner (31%), aunt (8%), uncle (23%), friend (54%), or other extended family (8%).

**Table 1:** Characteristics of young African American men who have sex with men participants (*N* = 25). For Likert scales, 0 was least and 10 was most

Characteristics	N (%)
Duration taking antiretroviral therapy (in years)	
Less than 1 year	3 (12)
1 to 2 years	7 (28)
3 to 5 years	7 (28)
More than 5 years	8 (32)
Employment status	
Full-time	5 (20)
Part-time	4 (16)
Unemployed	12 (48)
Other	4 (16)
Highest level of education	
10th grade or less	0 (0)
11th grade	3 (12)
12th grade	3 (12)
More than high school	18 (72)
Unknown	1 (4)
Self-reported ability to read	
Excellent	19 (76)
Good	4 (16)
Fair	2 (8)
Poor	0 (0)
Relationship status	
Single	12 (48)
Partnered	11 (44)
Married	1 (4)
Other	1 (4)
Has a close personal contact that currently helps remember to take medication	
Yes	13 (52)
No	12 (48)
Ever used the following drugs^a^	
Marijuana	9 (36)
Heroin	4 (16)
Cocaine	5 (20)
Meth or amphetamines	5 (20)
Inhalants	4 (16)
Used the following drugs within the past 6 months^a^	
Marijuana	10 (40)
Heroin	0 (0)
Cocaine	5 (20)
Meth or amphetamines	0 (0)
Inhalants	0 (0)
How many days per week alcohol is drunk^b^	
0	12 (48)
1–2	6 (24)
3–4	3 (13)
5 or more	3 (13)
Generally, how would you rank the communication between your healthcare provider and yourself? (Likert scale, median 9.5)^c^	
0–4	1 (4)
5–7	7 (29)
8–10	16 (67)
How comfortable are you that you generally know what you need to know about your HIV medication? (Likert scale, median 9.0)^c^	
0–4	0 (0)
5–7	6 (25)
8–10	(75)
How comfortable are you that you generally know about the possible side effects of your HIV medication? (Likert scale, median 8.5)^c^	
0–4	3 (13)
5–7	7 (29)
8–10	14 (58)

^a^Not mutually exclusive

^b^For those who drink, the median number of drinks per day was 4 (range 1–5)

^c^*N* = 24

### Acceptability

#### Acceptability of the intervention

Generally, the participants found the proposed intervention acceptable and useful. Concerning receiving a real-time reminder by text, there was appreciation for how it can overcome forgetting. Examples of how several participants positively reflected on the comment included, “I think it’s a good idea. It’s great. Sometimes I forget.” “I mean I think it is smart.” “I will do it. It’s a good idea.” “To get a text message or phone call would be awesome.” “The fact that you get a little notification, so it gives you extra notice if you missed the three days – whatever you are on. Your dad or somebody might contact you like, ‘What’s going on?’” It was also viewed as a way to help overcome a highly mobile lifestyle. “I always have my phone.” However, one participant cautioned, “If that can keep up with me, good luck! Seriously.” We learned from this participant and others that many of these men have active lifestyles visiting friends and family, going to bars and parties, sleeping over at someone else’s place, traveling, or otherwise moving around the city for other reasons.

Participants revealed that a current common practice to avoid missing medication when they were away from home was to take medication with them (pocket dose). “Like you said, there are instances when people get too busy and it may slip their mind. The best thing you can do for situations like that, if ahead of time you know that you are going to be busy, yes, put it in your pocket. That’s how we have been doing it for years. Put the bit in our pocket and go about our day.”

A few participants were not enthusiastic with at least one level of the triaged approach. One participant was ambivalent and distrustful of being monitored and sharing such data with a close contact. “It depended on who you let know about something like that. Like, you just couldn’t trust anyone with your business these days.” Another participant did not see the added value of a real-time text since they could set a reminder alert on their phone already, although their comment did not address that the intervention is in response to when they have forgotten rather than to alert them of the time to take it. “If you think about it, it doesn’t do anything. It’s just like my regular pill bottle. I get it has a light and it may send me a text. But on my phone, I can set an alarm to go off everyday at a certain time. That’s going to let me know to take my meds and I’ll hear that. This, I don’t see it catching my attention when I need to take my meds.” And another participant did not see the need for assistance with adherence, “I am a grown responsible man. If I don’t want to take my medication, I’m not going to take my medication. I wouldn’t want anybody interfering with my personal business like that. Period.” Finally, one participant was not confident the reminder would help him, “It’s funny because I get a text telling me when my bill is due and I still overlook it.” Some situations were brought up that could lead to missing where the proposed intervention would not overcome, such as being drunk or being out of town without their medicine.

### Privacy/stigma

Privacy was a major concern for participants especially with attention to possible disclosure of their HIV status by receiving a medication-related text that someone else might view and could lead to unwanted attention. “Your friends joke and say, ‘Somebody telling you you’ve got to take your medicine?’ that may make someone defensive.” One participant stated, “… It irks me … I get tired of trying to hide this over here...People are really nosy. Very curious.” One participant believed that disclosure of HIV status had led to his loss of a job. “I recently just had a job that I lost because my status was disclosed.” Similarly, another complained, “You just couldn’t trust anyone with your business these days. You know what I’m saying … That’s like I’m open to certain things like this whole situation but, like, I don’t know.” Another stated, “Sometimes people might do stuff and then it will leak it.” One participant said, “My family knows but I have some privacy … My business is not everybody’s business.” Finally, one stated, “I feel a lot of people, for those that are not in care, most of them are not in care because they are too worried about what everybody else is going to think or say about them if they found out. That’s why they don’t end up taking the medicine.”

Alternatively, there were participants who had no concern about privacy, for example, one participant stated, “I don’t have anything to hide. If it was to come across my phone and somebody saw it, it is what it is.” Another participant was not concerned about others knowing his status for fear of consequences as much as having to talk about it. “Privacy is a big issue for me … because I have zero to little tolerance for ignorance and stupidity …You really get to see how ignorant people are pertaining to the illness. Then you have to go and have a long spiel. For me to avoid all that, I hide my pills.”

Participants were most concerned with the content of the message rather than about getting a reminder on a cell phone. Regarding the choice of a text reminder, one participant said, “I don’t think it should say you missed your HIV medication,” and complained that he has had experience with someone “going through my phone.” Another offered, “As long as everybody uses a phrase or code that only they will understand, everything should work out fine.” Another echoed this point, “The selection of the wording would definitely play a tremendous part.” Most participants would use something cryptic, “…something that only I would get and nobody would get. Something so obscure and abstract…” Another stated, “I think it should be something professional and confidential if it is a message.” Examples of reminders that participants offered were diverse, readily offered, and reflected that they do not want the message to be obvious to others. “You know what time it is,” “Remember you have missed,” “You’re missing out on life,” “YOLO!” (*you only live once*), “Did you have your eight cups of water today,” “Don’t forget your vitamins,” “Skittles,” “Tic tacs,” “Bird seed,” “1, 2, 3” (*code for Atripla*), “Your pizza’s here,” and “Your beans are burning.” However, some participants did not care if the message was cryptic. One offered, “Hey this is reminding you to take your medicine,” as the text message. Another stated, “It wouldn’t even matter to me. Even with the text messages I have on my phone now. They’re very blunt and straightforward. I don’t care what the text message is because at the end of the day it’s not your life you have to live. It’s mine. What you have to say or feel about it, I don’t care.” Another participant agreed with this statement.

Alternatively, the idea of using an emoji was also put forth, “like a pill bouncing up and down. I think it should be funny, quirky, you know, corny-like. Like hey, people don’t want to feel pressured.” “Who doesn’t love emojis?” For example, participants suggested a flower, a hospital, and described a caduceus. “Maybe it can be like the security image where you pick whatever image you want it to be out of six or nine.”

Some participants expressed concern of taking medication from the device in a public space, “If I was to open this thing up …somebody that I know a little personally. Say, if I was opening it up and they see me taking the medicine. The first thing they are going to think is, ‘You got HIV?’” The physical design of the device was a concern for some participants because it could be confused with a taser. “Just recently in our community, if we were to be outside taking our meds or even if we just got pulled over and they asked ‘What is that in your pocket?’ That’s pitch black and looks like a tool.” Others stated, “That can end up getting you locked up.” “I thought it was a taser.” “It slides just like a taser would.” However, its resemblance to a taser was considered an advantage to one participant. “That might take away the stigma of, ‘What is that?’” Its appearance was also compared to an inexpensive cell phone. “The size is not a problem. Now that I look at it, this almost looks like a GoPhone**®**.” They liked that the device had a bland appearance because it would not be something that might get stolen.” A participant stated, “It looks nice.” The device’s bland appearance was an advantage. “I’m saying, people see this and they are never going to think this is a pill bottle. When I first saw it, I was like, ‘What is this? A phone?’ You would never know what it is.” One stated, “I believe it’s easy because other people wouldn’t think it would be a pill box. They’ll think it’s just a case. Therefore, you have an accessory.” “The concern for size revealed the participants would like to use the device as a portable way to have their pills with them. The rectangular shape was considered an advantage to transport because “normally the pill bottles are circular.”

### Feasibility

There were no concerns about inconvenience or difficulty of use. Despite clarification that the device was to replace their bottle (many kept their medication on their dresser at home), some raised concerns about what if they were carrying it on them. Several participants commented that it was larger than they would like whereas many were not concerned with its size. “It’s a good size. I could see where you could say it would be awkward because it’s big. Either way, for me it wouldn’t matter.” One participant also recommended that it come in different colors, another that it could be “sleeker” which would make it more portable. “I think it is too big, because if you are wearing some fitted or tight pants, that might pop out or look like you’ve got a pager.”

There were privacy concerns that were relevant to feasibility. Some participants wanted to know if the pills will make a rattling noise if they carried it around in their backpack or pocket since that would be a negative feature. One complained, “It is with that ch- ch- noise. Click clacking. Sounds like some rocks in your pocket… It draws attention. Like, ‘What is that? Tylenol? What do you have?’” Another added, “You don’t want to sound like a walking pharmacy.” Participants disclosed a variety of methods used to avoid this noise problem. These included putting a “tissue on it so it doesn’t make that sound” which could be suggested for the proposed intervention. Alternatively, as mentioned above, to avoid the shaking noise a dose could be put in a “little sack pocket” in their pants.

Concerning cell phone practices, texting was considered feasible but email or Facebook messaging were also suggested for the reminder. “If you got a Facebook account, you’re good! … If you download Facebook Messenger and your phone goes off, when you get wifi, you’ll still get your text messages.” “I know a lot of people who do not keep their numbers the same. If it wasn’t for Facebook, nobody would have contact with each other.” There was diversity of experience with the issue of changing cell phones. For example, participants stated, “I change phone companies, everything.” “I know some people that change their phone number like they change clothes.” “I have a new phone every month.” However, one participant stated, “I’ve had the same number for 20 years,” and another stated, “I ain’t never gonna have that problem. I don’t lose my phone. I don’t let anybody touch my phone.” Some had more than one phone and while it was offered that they would select one of them to receive the reminder, it was also offered that they would have all of them receive it.

#### Support persons

Family members were cited most often as the primary support person who they would volunteer for receiving notification of their two or three-day missed dose. “They know how to contact me constantly.” A mother was mentioned as a safe choice in three focus groups. Examples of participant comments included, “I know my mama’s not going to use this negative situation against me.” “She’ll get on me and I listen to my mother.” “I picked my mother because I trust my mother with my life.” “She’ll probably throw in, ‘Why did you miss it?’” Other persons mentioned included father, sister, brother, a female cousin, uncle, and aunt, “She has had the same cell phone number since 2003 until this very year … If something was to go wrong, I can count on her to be quicker than anybody else …I don’t have a mom or dad – they passed away.” As a back-up plan, one participant kept medicine at their aunt’s home. Among nonfamily members cited were a pastor, a friend, a co-worker, and their partner. “The person you would be contacting would be someone I’d be lying down, going to sleep with every day anyway. We’d be on each other about it because my partner is also positive as well. Every three days it switches up. ‘Did *you* take your medicine?’” One participant said he had no social support person.

Concerning a 7-day miss, most participants stated they wanted the alert to go to their case manager although some said their doctor and one said their pharmacist. Participants cited the doctors’ limited availability as a reason to prefer a case manager despite offering how much they trust and disclose sensitive information to their doctor.

Whereas most people did not have a problem with a trusted contact becoming involved in the intervention, some participants emphasized not choosing a contact and relying solely upon the computer-generated alert. Some participants acknowledged that the notification might lead to friction. “Of course I would get on it because it’s your mom, and you want to stay healthy and secure with that. But it would get on my nerves. It annoys me already. When I feel she is, ‘I see you still have a nice amount of’ ‘Get out of my business!’” “I don’t need my mom calling me and asking me questions. ‘Don’t lie to me.’” One participant did not want anyone to be contacted. “The computer is alright. A wrong person telling me, ‘Okay it’s time to take your medicine.’ I’m going to say--- Excuse my French, ‘Fuck you. You’re riding on my territory’.”

In summary and with consideration to the IMB model of antiretroviral therapy, receiving a text message that informs of missed doses in real-time was welcomed by most but needs to be self-selected and available not only by phone but also by back-up or alternative methods such as email or Facebook. Motivation to take a dose was expected by most participants either in response to a trusted social support person’s interaction when a multi-day dose was missed or in order to avoid the social support person getting notified because it could cause a negative feeling (as with regret for bothering them or concern for friction). Therefore, the combination of a real-time alert with a back-up involvement of a social support person was generally expected to promote the behavioral skill of taking medication. With consideration to the Technology Acceptance Model, most participants felt the intervention would be useful and the device was easy to use although enthusiasm for the device appearance was mixed and if modification of its appearance is feasible, it could be welcomed more by this population.

## Discussion

The purpose of the focus groups and one-on-one interviews was to inform the development of a triaged real-time alert intervention to improve treatment adherence among young AAMSM living with HIV. These focus groups provided information that helped to anticipate acceptability and feasibility, as well as to influence specific intervention features such as choice of text message reminders. The results demonstrate that young AAMSM living with HIV generally find the proposed intervention acceptable, but they had concerns especially about privacy. The focus groups also brought out potential challenges to anticipate with the social support stage of the triaged approach. The proposed triaged real-time alert is a promising approach for young AAMSM.

Regarding the acceptability of the proposed intervention, participants generally appreciated the reminder triggered by forgetting, giving them an opportunity to take the dose later the same day before a 24-h miss has occurred. Mobility was a common issue which some tried to overcome with pocket dosing or storing extra pills at an entrusted person’s home. Our intervention would alert in these circumstances. We expect this aspect of the intervention would not be a problem, but it did not come up if participants would consider it a benefit or annoyance in this situation.

Regarding privacy, a primary concern was that the alert text should not attract any unwanted attention to their health. In fact, several participants had code words for their HIV medication that they used with each other in public. In terms of intervention design, it became clear from the participants that the best course of action would be that each individual choose their own message and delivery mechanism (text or email) rather than a menu of possible messages be offered to them.

The bottle’s ability to attract attention was another concern. Some felt that it could lead to stigma if someone like a police officer mistook it for a taser and, in examining it, discovered and asked about their medication. Many participants thought the proposed intervention involved using the Wisepill device to carry around their medication, which led to comments about its size and appearance in public. However, as it is intended to replace their pill bottle that they commonly described keeping in the privacy of their home, we suspect this concern will not be a major feasibility issue. Concerns about device appearance or behavior are not new to acceptability studies of electronic adherence monitoring devices. For example, in Tanzania in a study of 23 persons living with HIV that used MEMS (a Medication Event Monitoring System device), participants appreciated that the bottle did not look like an ordinary medication bottle and thus disguised its true use [54]. The lack of a medication label was also considered a positive feature. Concerning shape, two participants volunteered that a flat shaped container would be preferred which is closer to the Wisepill device appearance than an ordinary medication bottle. Haberer et al. described a problem with acceptability of a different electronic monitoring device, Med-eMonitor, in persons living with HIV in San Francisco [55]. Med-eMonitor prompts users to take their medication with a chime. Although there was generally good acceptability to Med-eMonitor (e.g., 66% of the 52 patients studied would “likely use the device again”), 65% of the patients stated the chimes were “annoying.” Auditory problems were not an issue in our study because the Wisepill device makes no noise. If Wisepill triggers a text message, the user can choose to silence their phone.

Another important issue impacting feasibility is cell phone access. Ninety-five percent and 98% of US adults and African-Americans own a mobile phone, respectively [56]. Cellphones are commonly with or near the owner. However, some of our participants described changing cell phones. Other work we have performed in this community has demonstrated that, for those who have low socioeconomic status, loss, theft, or lack of funds to maintain service can be a problem [unpublished data]. Interruption of cell phone access could definitely limit the potential effect of the intervention, especially if for a prolonged period of time. However, if someone has a working cell phone, even without service, they may receive email and possibly text messages whenever they gain access to free Wi-Fi. Retaining the same number or notifying the Wisepill server of the change can mitigate the challenge of changing cell phones. We anticipate that loss of participant contact information will be a problem for a minority of persons in this intervention, but may be a significant challenge over time. This problem might be overcome during a clinical trial with use of periodic financial incentives and outside of a trial with periodic server sent text messages reminding participants to update the intervention coordinator if they expect a change in phone number.

Another important issue to anticipate concerns the support person level of triage. The selection of support persons was diverse and even extended beyond family members or partners/spouses. Participants generally welcomed a trusted person’s involvement in the intervention scheme. We suspect that since their involvement would be limited to 2- or 3-day misses, for many this would keep such notification to a minimum. However, there is the possibility that such notification could stress a relationship if it is recurrent. Atukunda studied 62 people living with HIV and 41 social support persons in a study that included real-time adherence monitoring and two types of text messaging intervention in Uganda [57]. This study reported that nearly one-third of the social supporters perceived the support they provided to their respective HIV-positive study participants as discouraging and 24% reported that they were not happy in their relationships with their study participant at exit. Any stress that results from social support person notification and response may have a positive or a negative effect on adherence, but we suspect that it will be a negative motivator because some participants may take their medication to avoid the interaction. Based on our participant background information, more than two-thirds either had or could identify a social support person for the intervention. That still leaves almost one-third that may not identify anyone. Whereas this could limit the efficacy of the intervention, a case manager might assume the notification role of a 3-day and a 7-day miss. If the case manager reaches out in response to a 3-day miss, it could lead to early recognition of remediable adherence issues such as loss of insurance, mental health concerns, or provider failure to provide refills – all of which the case manager could try to assist.

A potential limitation of this study is participation bias. Some young AAMSM living with HIV may have been reluctant to participate in a focus group where their HIV status would become known to others present. Those who were willing to participate might have had different behaviors or opinions about the intervention than those who did not respond to the recruitment methods. This bias was not measurable. Another limitation is the small sample size which may limit generalizability. Also, although the moderator reassured the participants to feel comfortable sharing their thoughts, participants may have been uncomfortable sharing fully with others present.

## Conclusions

A triaged real-time alert intervention to improve treatment adherence is acceptable and feasible among young AAMSM living with HIV. Our next step with this work is to provide this real-time monitoring device to young AAMSM living with HIV to monitor their adherence for 3 months and contact them when their first 1 day, 3 day, and 7 day miss occurs to determine real-time reasons for missing and gather additional acceptability and feasibility issues to inform a randomized clinical trial of the intervention.

## Abbreviations

AAMSM: African-American Men who have Sex with Men
ART: Anti-retroviral therapy
COIP: Chicago Community Outreach Intervention Project
IMB: Information, Motivation, Behavioral skills
